# Learning Outcomes of e-Learning in Psychotherapy Training and Comparison With Conventional Training Methods: Systematic Review

**DOI:** 10.2196/54473

**Published:** 2024-07-29

**Authors:** Kasperi Mikkonen, Eeva-Eerika Helminen, Samuli I Saarni, Suoma E Saarni

**Affiliations:** 1 Faculty of Medicine University of Helsinki Helsinki Finland; 2 Department of Psychiatry Helsinki University Hospital Helsinki Finland; 3 Faculty of Medicine and Health Technology University of Tampere Tampere Finland; 4 Psychiatry Wellbeing Services County of Päijät-Häme Lahti Finland

**Keywords:** mental health, psychotherapy, digital learning, Kirkpatrick model, e-learning, online health, psychotherapy training, learning outcome, learning outcomes, systematic review, training methods, mental disorders, mental disorder, accessibility, evidence-based, scalability, cost-effectiveness, internet, education

## Abstract

**Background:**

Mental disorders pose a major public health problem in most western countries. The demand for services for common mental health disorders has been on the rise despite the widespread accessibility of medication. Especially, the supply and demand for evidence-based psychotherapy do not align. Large-scale increase of modern psychotherapy is difficult with current methods of training which are often expensive, time consuming, and dependent on a small number of top-level professionals as trainers. E-learning has been proposed to enhance psychotherapy training accessibility, quality, and scalability.

**Objective:**

This systematic review aims to provide an overview of the current evidence regarding e-learning in psychotherapy training. In particular, the review examines the usability, acceptability, and learning outcomes associated with e-learning. Learning outcomes are assessed in different modalities including trainee experiences, knowledge acquisition, skill acquisition, and application of trained content in daily practice. Furthermore, the equivalence of web-based training and conventional training methods is evaluated.

**Methods:**

Following the PRISMA (Preferred Reporting Items for Systematic Reviews and Meta-Analyses) guidelines, a search from Ovid, MEDLINE, PsycINFO, and Scopus databases between 2008 and June 2022 was conducted. Inclusion criteria required studies to describe e-learning systems for psychotherapy training and assess acceptability, feasibility, or learning outcomes. The risk of bias was evaluated for both randomized and nonrandomized studies. Learning outcomes were categorized using the Kirkpatrick model. Effect sizes comparing e-learning and traditional methods were calculated.

**Results:**

The search yielded 3380 publications, of which 34 fulfilled the inclusion criteria. Positive learning outcomes are generally associated with various e-learning programs in psychotherapy training including trainee satisfaction, knowledge, and skill acquisition, and in application of trained content in clinical practice. Learning outcomes generally show equivalence between e-learning and conventional training methods. The overall effect size, indicating this disparity, was 0.01, suggesting no significant difference. This literature displays a high level of heterogeneity in e-learning solutions and assessment methods.

**Conclusions:**

e-Learning seems to have good potential to enhance psychotherapy training by increasing access, scalability, and cost-effectiveness while maintaining quality in terms of learning outcomes. Results are congruent with findings related to e-learning in health education in general where e-learning as a pedagogy is linked to an opportunity to carry out learner-centric practices. Recommendations for conducting psychotherapy training programs in blended settings supported by activating learning methods are presented. However, due to the heterogeneity and limitations in the existing literature, further research is necessary to replicate these findings and to establish global standards for e-learning, as well as for the assessment of training outcomes in psychotherapy education. Research is especially needed on the effects of training on patient outcomes and optimal ways to combine e-learning and conventional training methods in blended learning settings.

## Introduction

Mental disorders pose a global public health problem [1]. Psychotherapy has been found to be effective across a variety of mental disorders [2-4]. However, access to evidence-based psychotherapy in public health care settings is often overly difficult and the supply and demand of evidence-based methods do not align [5]. One widely accepted strategy in meeting the existing and increasing demand of mental health services is to train health care professionals in various psychotherapies. The need for training encompasses both the training of new practitioners and updating the competences of existing professionals following the approach of continuing medical education.

Closing the gap between the demand for services and available resources through conventional training methods can be challenging. The main barriers in the dissemination of psychotherapy training include cost and time [6]. Conventionally, psychotherapy training is labor-intensive and relies heavily on the time provided by top-level professionals carrying out lectures and workshops that often lead to expensive and time-consuming programs and problems when striving to scale up. While psychotherapy training is linked to positive learning outcomes, the evidence on the impact of training on patient outcomes is mixed [7]. Digital solutions and e-learning have been acknowledged as a potential tool in expanding the reach of training, increasing the quality of training programs, and improving the dissemination of treatment [8-10]. With the comprehensive educational need, using digital solutions may be necessary.

e-Learning, which is often used synonymously with “digital learning,” “online learning,” and “web-based learning,” can be conceptualized as a framework that combines educational theories and technology or a practical application of educational technology that evolves in tandem with the advancements in technology and the internet [11,12]. The terminology is overlapping, and different labels give rise to diverse expectations [13]. One detail in terminology to highlight is the emphasis on “distance” between the trainer and the trainee, which is not a defining characteristic of e-learning [14]. It is tied to the trainee engagement and whether the training is held synchronously (eg, Zoom lectures) or asynchronously (eg, self-directed and individually paced learning in a web-based environment with multimedia materials and assignments).

Asynchronous e-learning with an emphasis on the active role of the trainee offers a possible solution for many current problems in psychotherapy training. First, e-learning offers a platform to carry out pedagogical principles that prioritize trainee engagement, as seen in approaches such as the flipped classroom, which involves a transition from trainer-led lectures to individual studying which happens before the synchronous learning event and which has been associated with improvements in learning outcomes compared with conventional training methods in health profession education [15,16]. Second, asynchronous e-learning offers flexibility in terms of time and place, which can remove some of the constraints involved in conventional training methods. Third, e-learning content can be scaled up more easily than conventional training methods. Fourth, while the current training programs are mostly rooted on various therapeutic traditions (or “schools”), which often hinders evidence-based development of psychotherapy, an adequate use of e-learning can be a way to create transparent training content which is easy to update and which fosters therapeutic competence [17-19]. Moreover, while the evidence regarding the cost-effectiveness of e-learning in health education is still in its infancy, e-learning is often associated as a means to increase the cost-effectiveness of training programs in the long term [20].

e-Learning is a widely used method for training in various industries and is generally associated with positive learning outcomes that are either comparable or superior to conventional training methods [21-23]. The literature examining e-learning in psychotherapy training is emerging, and its promise has been recognized [24]. However, an overview of the current evidence-base remains undetermined, and it remains unspecified how e-learning should be precisely used. For example, what is the role of e-learning in knowledge and skill acquisition, and is the value of e-learning most apparent when implemented as a blended learning curriculum, which combines conventional training methods with e-learning [19]?

The goals of this systematic literature review are to (1) provide an overview and assess the quality of the current literature; (2) determine the acceptability, usability, and achievable learning outcomes of e-learning in the context of psychotherapy training; and (3) assess how does e-learning compared with conventional training methods. Based on our results, we aim to formulate the current maturity and limitations of e-learning in this domain and discuss what kind of recommendations can be made regarding e-learning and psychotherapy.

## Methods

### Design

A systematic review was conducted to extract recently published scientific papers that dealt with e-learning in psychotherapy training. We focused on the last 15 years (from 2008 onward) since rapid software and hardware development has occurred during that period although internet delivered and computerized e-learning as a concept emerged as early as the 1990s [25]. This review followed the PRISMA (Preferred Reporting Items for Systematic Reviews and Meta-Analyses) guidelines [26].

### Search Method

The search was conducted within Ovid MEDLINE, PsycINFO, and Scopus databases. The search strategy included a process where overlapping labels depicting psychotherapy and e-learning were combined. The search was formulated to cover both the training of larger treatment modalities and the specific therapeutic competences. Thus, the search strings were as follows: (psychotherap* or psycholog* or therap* or counsel*) adjacent to (skill* or train* or educat* or compentenc*) OR (interpersonal) adjacent to (skill* or train* or educat* or compentenc*) OR (psychotherap* or psychosoc* or mental health or behavioral health or behavioural health) adjancent to (intervent* or treat* or therap* or evidence-based intervent* or evidence-based treat* or evidence-based therapy* AND (digital* or mobile or web-based or online or distance) adjancent to (learn* or train* or educat* or program* or self-administered learn* or self-administered train* or self-administered educat* or self-administered program*) OR (e-learn* or elearn* or e-educat*). The searches were conducted on all databases in June 2022. To ensure optimal coverage considering the field’s unestablished terminology, a comprehensive array of search terms was used.

### Selection Criteria

#### Inclusion

To be included in this review, the studies should involve (1) a description of an asynchronous e-learning system (ie, a digital learning environment, eg, a website designed for self-directed learning) in the context of training psychotherapy competences and (2) measures of acceptability, feasibility, or learning outcomes.

#### Exclusion

Studies were excluded if they (1) represented an unpublished thesis or dissertation, (2) were not peer-reviewed, (3) were not experimental studies, (4) were unavailable in English, and (5) defined e-learning solely as synchronous distance learning (eg, Zoom lectures).

#### Study Selection

The eligibility of the search results was assessed by the first (KM) and last (SES) authors through three sequential steps: (1) initially, KM meticulously examined the titles and abstracts of all search results, systematically eliminating studies that distinctly met the specified exclusion criteria; (2) in cases where the title or abstract lacked sufficient detail to determine eligibility, KM proceeded to evaluate the full text of the study, applying the same exclusion benchmarks as in the initial phase; and (3) for the final step, KM scrutinized the full texts of all remaining studies, while SES independently reviewed a randomly selected subset of these studies to ensure representativeness. Any differences in opinion were subsequently resolved through a collaborative discussion targeting a unanimous decision. This stepwise approach ensured that studies were disqualified only if they unambiguously satisfied the predefined exclusion criteria. Given our anticipatory search terms’ breadth, a substantial volume of studies not aligning with our inclusion criteria, such as those investigating digital treatment interventions rather than the training of professionals, was anticipated. Hence, to optimize efficiency, the first 2 screening stages were singularly executed by 1 reviewer (KM).

### Data Collection

Examination of the titles and abstracts of the publications and the assessment for potential inclusion were carried out by first (KM) and last author (SES). Data extraction was carried out by the first author and included (1) target group of the training; (2) description of training content; (3) description of e-learning methods; (4) comparison between training modalities; (5) sample sizes; (6) primary outcome; and (7) summary of results. Furthermore, to calculate a measure of effect size for comparing differences between conventional training methods and e-learning, the means and standard deviations of the learning outcomes reported in the studies were extracted.

### Risk of Bias

The risk of bias was assessed with the latest Cochrane risk-of-bias tools: RoB 2—tool for randomized trials and ROBINS-I (Risk of Bias in Non-randomized Studies of Interventions) for nonrandomized trials [27,28]. The risk of bias analysis was systematically conducted by the first (KM) and last (SES) authors in an independent manner. Subsequent to this individual assessment, any emergent discrepancies were collaboratively addressed through discussions focused on achieving consensus.

### Framework for Classifying Results

We classified the results of the primary outcomes of the included studies using the Kirkpatrick model of training evaluation [29], which evaluates training outcomes on 4 levels. The first level (“reaction”) evaluates the training participants’ immediate reaction and feedback regarding the training program including satisfaction, engagement, and relevance. The second (“learning”) evaluates the learning outcomes of the training program including change in attitudes, confidence, knowledge, and skills. The third level (“behavior”) evaluates how much the trainee participants apply the learned knowledge and skills when treating patients in real life. The final level (“results”) evaluates how much the patient outcomes improve because of the training.

### Comparison Between Training Methods

In order to quantify the magnitude of the difference observed in these results, Hedges *g* was calculated as a measure of effect size with 95% CIs [30]. Hedges *g* is comparable with Cohen *d* but corrects small sample bias. Therefore, Hedges *g* of 0.2 represents a small effect; 0.5, a moderate effect; and 0.8, a large effect [31]. The effect size was calculated from the studies that included the figures necessary for the calculations. For studies with missing information, the first author of the publication was contacted, and they were asked for the data. An overall effect size describing the equivalence between training methods was formulated as an arithmetic mean of all calculated effect sizes.

## Results

### Search Outcome

**Figure 1:**
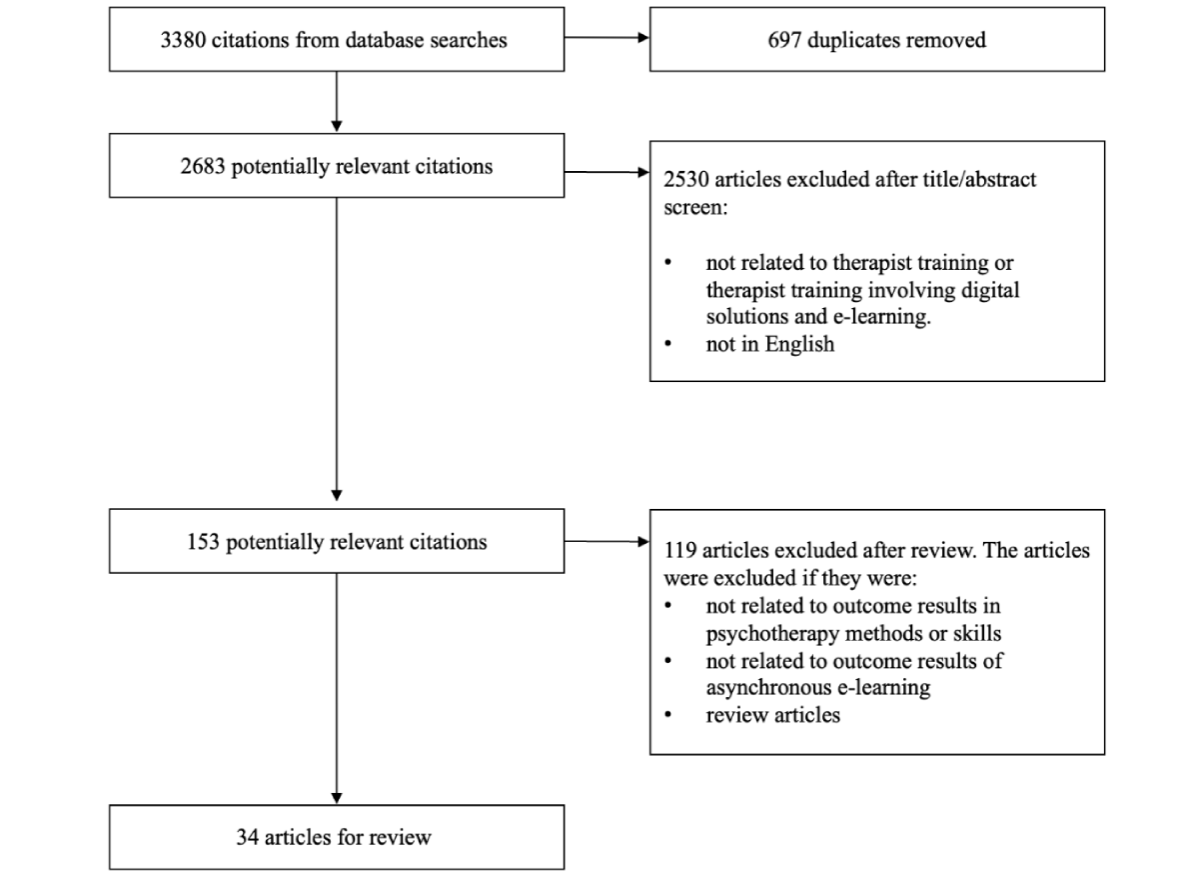
PRISMA (Preferred Reporting Items for Systematic Reviews and Meta-Analyses) flowchart.

The first screening from the databases identified 3380 articles. Duplicates (n=697) were removed using the “Merge items” tool in the “Duplicate items”—folder in “Zotero,” which the authors used as a reference management software. After removing the duplicates, 2683 articles were identified and the eligibility was assessed by KM based on their title, and abstract if necessary, and 2530 articles were excluded as clearly fulfilling the exclusion criteria mentioned in the “Selection Criteria” section of the article (see Figure 1). The remaining 153 articles were read in full-text versions by KM and 79 articles were excluded according to the same criteria. KM reviewed the remaining 74 articles in full, and SES reviewed in full, a random sample of those articles (n=45). The size of random sample required was calculated using Cohen κ for interrater agreement level with the assumptions that 30%-40% of the remaining articles would be included in the final sample, and the interrater agreement is moderate at the least [32]. The calculations were conducted with R with the “CIBinary”—function within the “kappaSize”—library. Based on these calculations, the last author reviewed the random sample of 45 articles. The authors worked blind to each other. The results were compared, and the authors were in substantial agreement on which articles to include and exclude (Cohen κ=0.73). All discrepancies were discussed to a consensus (eg, a detailed discussion whether the target of a training described in a specific study was related to psychotherapy skills or more general interaction skills used in health care). Thirty-four studies were included in the final review.

The articles included in the study are listed in Table 1 for randomized controlled trials (n=17) and Table 2 for nonrandomized (n=17) studies. The tables include the target group of the training, the content of the training, a depiction of the e-learning system used, a comparison between training modalities if applicable, the primary outcomes of the studies, and a summary of the main findings. In all studies, training was targeted to health care providers. The training content was most often related to some form of cognitive behavioral therapy (CBT) and the length of the e-learning programs ranged from 15 minutes to 52 hours. In the studies included, the effects of e-learning are evaluated in within-trainee designs and comparisons with waiting for training and other active training methods. The data were extracted by the first author as they were presented in the original studies.

**Table 1:** Randomized controlled trials included in the review.

Study	Target group	Training content	Description of e-learning	Comparison and sample sizes	Primary outcome	Summary of results
Shinohara et al (2022) [33]	Midwives and public health nurses	Perinatal mental health assessment and empathic communication	1-day course with short video scripts	Intervention group which received training (n=58) vs no-training control group (n=57)	Improvement of empathic communication	The intervention group was at a significantly higher level for empathic communication skills compared with the control group (*P*<.001)
Nisar et al (2022) [34]	Nursing school students	Evidence-based psychosocial intervention for perinatal depression	A tablet-based, multimedia training program including narrative scripts	e-Learning intervention group (n=42) vs conventional specialist–delivered training (n=47)	Competence in the trained intervention posttraining and at 3-month follow-up	e-Learning was not inferior to specialist-led training (*P*=.14)
Taylor et al (2021) [35]	Licensed behavioral health providers	Cognitive Behavioral Therapy for Insomnia	A website including video instructions and demonstrations, descriptions of intervention techniques, handouts, and exercises	e-Learning group (n=21) vs in-person workshop control (n=23)	Knowledge acquisition in CBT-I ^a^	Knowledge acquisition was equivalent in both groups (*P*=.16)
Sansen et al (2020) [36]	Psychotherapists and psychotherapist trainees	Trauma-focused cognitive behavioral therapy	52-hour web-based training course including written information, animations, video clips, various assignments, and simulated cases	e-Learning group (n=247) vs waitlist control group (n=252)	Subjective ratings of knowledge, confidence, competencies, and willingness to use trauma-focused intervention and performance in a knowledge test	Significantly greater improved subjective ratings of knowledge, confidence, competencies, and willingness, as well as performance in the knowledge test in the e-learning group compared with the control group (all *P* values <.01)
Sansen et al (2019) [37]	Psychotherapists and psychotherapist trainees	Trauma-focused cognitive behavioral therapy	52-hour web-based training course including written information, animations, video clips, various assignments, and simulated cases	e-Learning group (n=247) vs waitlist control group (n=252)	Fears and reservation toward conducting trauma-focused methods	A significant reduction of fears and reservations during e-learning (*P*<.01)
Kullberg et al (2020) [38]	Undergraduate psychology students	Suicide prevention practice	1-hour web-based training including videos of professionals interacting with various patients with suicidal tendencies	e-Learning group (n=211) vs waitlist control group (n=187)	Self-reported guideline adherence, knowledge acquisition, and provider confidence	Significantly greater improvements in self-reported knowledge, confidence, and guideline adherence in the e-learning group compared with the control group (all *P* values <.01). Learning effects were maintained in 3-month follow-up
Rahman et al (2019) [39]	Nonspecialist health workers	Psychosocial management of perinatal depression	A tablet-based training course including narrative scripts, reflective assignments, and video role-plays	e-Learning group (n=30) vs face-to-face training control group (n=30)	Competence in intervention	No differences between e-learning and face-to-face training in competence scores post assessment and at 3-month follow-up (*P*>.05)
Puspitasari et al (2017) [40]	Mental health clinicians	Behavioral activation	4 × 40-minute web-based audio-guided presentations of core BA^b^ skills	e-Learning intervention group- (n=40) vs trainer-led training (n=37)	BA skills competence	Significantly greater increases in all BA skills in trainer-led training compared with web-based training (*P*<.0005)
Stein et al (2015) [41]	Mental health clinicians	Interpersonal and social rhythm therapy for bipolar disorder	12-hour self-paced web-based training	e-Learning group + 3-6 months of monthly telephone supervision (n=16) vs control group in 2-day workshop and weekly supervision (n=20)	Use of IPSRT^c^ techniques during treatment	A significant increase in the use of IPSRT techniques in posttraining which increased in 12-month follow-up (*P*<.01). The increase was comparable in both groups
Dimeff et al (2015) [42]	Mental health clinicians	Two core strategies in DBT^d^: chain analysis and validation	Interactive, media-rich web-based training modules with expert commentaries, exercises, simulations, printable handouts, and knowledge checks	Web-based training (e-learning) (n=55) vs ILT^e^ (n=55) vs TM^f^ (n=62)	Satisfaction, self-efficacy, motivation, knowledge, clinical proficiency, and clinical use	ILT was superior compared with web-based training (e-learning) and TM in satisfaction (*P*<.0001), self-efficacy (*P*<.001), and motivation (*P*<.05). Web-based training (e-learning) was the most effective method for increasing knowledge (*P*<.01). There were no differences between observer-rated proficiency (*P*=.76) and self-reported clinical use (*P*=.48), which increased during training
Harned et al (2014) [43]	Mental health providers	Exposure therapy for anxiety disorders	10-hour course incorporating gaming technology and evidence-based instructional design strategies. The learner could choose whether to engage with media-rich didactic materials or simulated clinical scenarios	Web-based training (e-learning) (n=60) vs web-based training (e-learning) + brief computerized motivational enhancement intervention (web-based training [e-learning] + ME^g^) (n=60) vs web-based training (e-learning) + ME + a web-based learning community (web-based training (e-learning) + ME + LC^h^) (n=61)	Knowledge acquisition, attitudes toward exposure therapy, self-efficacy, clinical use, and clinical proficiency	All training methods comparably improved self-efficacy and clinical use of exposure therapy. The increase in attitudes in the web-based training (e-learning) + ME + LC group at 6-week follow-up was significantly larger than in the web-based training (e-learning) group (*P*=.01) as well as in clinical proficiency at 12-week follow-up (*P*=.04). Web-based training (e-learning)–ME-LC condition was also superior in knowledge acquisition (*P* values =0.004-0.05)
Ruzek et al (2014) [44]	Mental health clinicians with responsibilities in treating veterans with PTSD^i^	CBT^j^ skills for traumatic stress: motivation enhancement, goal setting, and behavioral task assignment	3-module web-based training with interactive exercises, skills demonstrations, downloadable materials, and bibliographies	e-Learning group (n=46) vs e-learning + telephone consultation (n=42) vs no-training or training as usual (free to take part in any training activities they would otherwise receive) (n=51)	Skills acquisition	Skills improved significantly in both active training groups compared with the control group in motivation enhancement and goal setting (*P*<.001, *P*<.005). Web-based training plus supervision was superior compared with web-based training alone in the motivation enhancement module (*P*=.001) with no significant differences in the 2 other modules.
Larson et al (2013) [45]	Addiction counsellors and supervisors	Cognitive behavioral therapy	Web-based training including various exercises, audio-vignettes and role-played treatment sessions, and an assessment between modules	e-Learning (n=47) vs training manual (n=52)	Adherence to CBT practice	No statistically significant difference in pass rates and adequacy scores in adherence to CBT practice between groups (*P*=.19). The posttraining gains in CBT skill adequacy were small
Bennett-Levy et al (2012) [46]	Practicing in a counseling and mental health role with a relevant degree or students closely completing a degree	Cognitive behavioral therapy	30-module web-based training with didactive text and video demonstrations with case examples and quizzes	e-Learning group (n=23) vs supported training group with the possibility of six 15-minute support calls (n=24)	Completion rate, knowledge acquisition, self-reported skills acquisition, confidence in CBT, and skills use in practice	Both groups demonstrated similar improvements in objective knowledge test and on self-reported measures of knowledge, skills, confidence, and use of skills at follow-up (improvement all significant at *P*<.001). Supported training group had a significantly higher completion rate (*P*<.05).
Dimeff et al (2011) [47]	Community mental health providers	DBT distress tolerance skills	A 5-module web-based training with media-rich content, expert insights, practice exercises, and knowledge checks with downloadable handouts	e-Learning (n=47) vs training manual (n=43) vs placebo e-learning (n=42)	Knowledge acquisition, self-reported self-efficacy, and motivation to use learned skills	Web-based training and the TM outperformed the control condition in knowledge acquisition (*P*<.05) and self-efficacy (*P*<.05) but not in motivation to learn and use the treatment. By the 15-week follow-up, the web-based training outperformed manual in knowledge acquisition (*P*<.05)
Dimeff et al (2009) [48]	Community mental health providers	Dialectical behavior therapy	A 20-hour 5-module web-based training with media-rich content, expert insights, clinical vignettes, practice exercises, and knowledge checks	e-Learning (n=54) vs training manual (n=49) vs ILT (2-day workshop) (n=47)	Satisfaction, knowledge acquisition, confidence, self-efficacy, and application of trained content. Performance in structured role-play	Higher satisfaction in web-based training and ILT than in training-manual (*P*<.001), but no differences between web-based training and ILT (*P*=.33). Web-based training outperformed the other conditions in knowledge acquisition (*P*<.05) while there were no differences between ILT and training manual (*P* values >.90). At posttraining, web-based training and ILT conditions reported more elevated self-efficacy than the manual condition (*P*<.01) but the differences diminished in follow-up. All training methods resulted in comparable increase in the application of trained content in clinical situations (*P*=.68). Adherence and competence in role-play scenario improved significantly in all conditions but there was no difference between conditions (*P* values >.20)
Weingard et al (2006) [49]	Substance abuse counsellors	A cognitive behavioral approach in treating cocaine addiction	A 60-minute web-based training module with pre- and posttests	e-Learning (n=52) vs face-to-face workshop (n=55) vs delayed training (n=59)	Knowledge acquisition	Improvements in knowledge were significant comparable in both web-based training and face-to-face workshops

^a^CBT-I: Cognitive Behavioral Therapy for Insomnia.

^b^BA: behavioral activation.

^c^IPSRT: interpersonal and social rhythm therapy for bipolar disorder.

^d^DBT: dialectical behavior therapy.

^e^ILT: instructor-led training.

^f^TM: treatment manual.

^g^ME: motivational enhancement.

^h^LC: learning community.

^i^PTSD: posttraumatic stress disorder.

^j^CBT: cognitive behavioral therapy.

**Table 2:** Nonrandomized trials included in the review.

Study	Target group	Training content	Description of e-learning	Comparison and sample size(s)	Primary outcome	Summary of results
Wilkerson et al (2022) [50]	Health and mental health professionals, educators, and trainees	Cognitive Behavioral Therapy for Insomnia	6- to 10-hour 10-module web-based training with didactic videos, therapy vignettes, and resources regarding the specific modules	N=2586	Training completion rate and knowledge acquisition	1203/2586 (46.52%) of registered users started the training and 570/2586 completed the training with a 3-month period with both pre-and posttests complete. A significant increase in knowledge was reported (*P*<.001)
Seidler et al (2022) [51]	Mental health practitioners	Engaging men in therapy and dealing with mental distress and suicidality experiences by males	8-hour web-based media-rich asynchronous training program including written information, downloadable worksheets, and videos	N=192	Improvement in self-reported competencies in engaging male clients	Significant increase in self-reported competencies (*P*<.001)
Soll et al (2021) [52]	Postgraduate health care professionals	Cognitive behavioral therapy for psychosis	Prerecorded lectures, exercises, web-based role-plays, and web-based conferences	e-Learning (n=85), in-person training (n=142)	Satisfaction and acceptance	Participants’ satisfaction and acceptance toward the web-based training were noninferior compared with in-person training in all respects except for “room for active participation” and “professional benefit”
Applebaum et al (2021) [53]	Health care professionals working in psychosocial oncology	Acute cancer cognitive therapy	6-hour web-based training course including written information and various assignments	N=46	Knowledge acquisition	A significant increase in knowledge (*P*<.001)
Gonzalez Salas Duhne et al (2020) [54]	Professionals delivering psychological intervention	Implementation intentions (“if-then”—planning)	A 15-minute video-based training program with pre- and postquestionnaires	N=87	Knowledge acquisition, acquisition of practical knowledge, and self-reported use of implementation intentions	Significant increase in knowledge both posttraining and in 1-month follow-up (*P*<.001). No significant increase in practical knowledge. A significant increase in use of implementation intentions (*P*=.018)
Graber (2019) [55]	Psychiatric mental health nursing students	Therapeutic crisis management techniques	A 30-minute presentation in a web-based educational environment	e-Learning (n=47), in-person training (n=63)	Knowledge acquisition and student satisfaction	No differences between e-learning and in-person training in knowledge acquisition (*P*=.483) or student satisfaction (*P*=.87)
Cramer et al (2019) [56]	Mental health professionals	Core competencies in suicide prevention	20-hour self-paced narrated presentations with associated resources and handouts, case descriptions, and various assignments	N=43	Knowledge acquisition, attitudes toward self-harm patients, and skills performance	Improvement in knowledge (*P*<.001), perceived skill mastery (*P*<.001), capacity to work with suicidal patients (*P*<.001), objective risk judgment accuracy (*P*<.001), and reduction of negative feelings about self-harming patients (*P*<.007)
Meredith et al (2018) [57]	Nurses and occupational therapists in mental health settings	Sensory modulation approaches in mental health	A 2-hour, 3 self-paced modules including interactive activities, case scenarios, opportunities of self-reflection, and links to further reading	N=121	Knowledge acquisition and change in perceived level of knowledge confidence and attitudes	Significant increase in knowledge acquisition, confidence, and attitude toward sensory modulation approaches (all *P* values <.001)
German et al (2018) [58]	Mental health clinicians	Cognitive behavioral therapy	A self-paced web-based training including videotaped role-playing, on-screen activities, and quizzes	e-Learning with peer-led consultation (n=148), in-person training with expert-led consultation (n=214)	CBT^a^ competency, knowledge acquisition, and learner retention	e-Learning with peer-led consultation was not inferior to in-person training with expert-led consultation in increasing clinical competency. No differences between knowledge acquisition between groups. Trainees in the web-based training program were less likely to complete the training (*P*<.001)
Kuhn and Hugo (2017) [59]	Psychiatry residents	Prolonged exposure to posttraumatic stress disorder	A 7- to 8-hour 13 web-based modules including step-by-step instructions, demo videos of expert clinicians, printable scripts, pre- and posttests, and a “common questions” section	N=12	Trainee impressions	The web-based training used in a blended training program may help address the common problems in traditional training models. The first impressions of the program support its viability, practicality, and efficiency
Kobak et al (2017) [60]	Mental health clinicians	Interpersonal therapy	A 4-hour web-based training with animations, graphical illustrations, clinical vignettes, and various exercises	N=26	Knowledge acquisition	Significant increase in knowledge of interpersonal therapy (*P*<.001)
Fairburn et al (2017) [61]	Mental health clinicians	Cognitive Behavioral Therapy for Eating Disorders	A 9-hour web-based training providing information on how to implement CBT-E^b^ with videos, handouts, learning exercises, and tests of knowledge with feedback. A separate digital library of detailed materials of therapy implementation with various subgroups	N=80	Competence in CBT-E	A significant increase in competence scores after completing the training (*P*<.001). Of the trainees 42.5% reached a predetermined cutoff point indicating good competence
Curran et al (2015) [62]	Counsellors working with individuals with substance use disorders	Manualized CBT group treatment for depression	A 12- to 16-hour media-rich and interactive digital training	N=7	Feasibility of self-paced digital training in replacement of in-person training. Observed barriers and facilitators, recommendations for improvement, and concerns of implementation	The main barrier concerning the completion of the training was the lack of protected time. The main facilitators included positive attitudes toward the training, supervisor support, counselor dedication, and beliefs supporting providing services for depression
Hickey et al (2015) [63]	Psychiatry residents	Two core topics in IS-TDP^c^	Two asynchronous e-learning modules of “the metapsychology of the unconscious” and “Davanloo’s technique of IS-TDP”	e-Learning (n=19), in-person training (n=19)	Knowledge acquisition	Significant improvement in knowledge levels during training in both topics (*P*<.01). No differences between knowledge acquisition between web-based training and lectures (*P*<.05)
Samuelson et al (2014) [64]	Primary care providers	Detection, assessment, initial management, and referral of patients with PTSD^d^	70-minute web-based training including multimedia didactic content, case presentations, and clinical vignettes	N=73	Knowledge acquisition, comfort regarding PTSD-related skills, and usage in clinical practice	Significant increase in PTSD-related knowledge (*P*<.001) and significantly greater comfort regarding skills compared with pretraining (*P*<.001). 47% reported using the training content in clinical practice
Kobak et al (2013) [65]	Social workers, psychologists and social work, and psychology students	CBT for anxiety disorders	A 5-hour 10-module web-based training with interactive exercises, animations, graphical illustrations, and expert demonstration videos	N=39	Knowledge acquisition and satisfaction	A significant increase in knowledge in CBT (*P*<.001) and trainees were satisfied in both the content and the technical aspects of the web-based training
Martino et al (2011) [66]	Counsellors working with substance use disorders	Motivational interviewing	Four 1-hour modules with instructions for self-practice with reflections on a discussion board managed by a course instructor	N=26	Adherence and competence in motivational interviewing	The web-based training was a part of a stepwise training program with workshops and supervision after the web-based training which were offered only if the learner showed inadequate competence after the web-based training. The participants who performed adequately after the web-based training did not improve in subsequent training. Participants who showed inadequate competence after the web-based training continued to improve in subsequent training

^a^CBT: cognitive behavioral therapy.

^b^CBT-E: Cognitive Behavioral Therapy for Eating Disorders.

^c^IS-TDP: intensive short-term dynamic psychotherapy.

^d^PTSD: posttraumatic stress disorder.

### Risk of Bias

**Figure 2:**
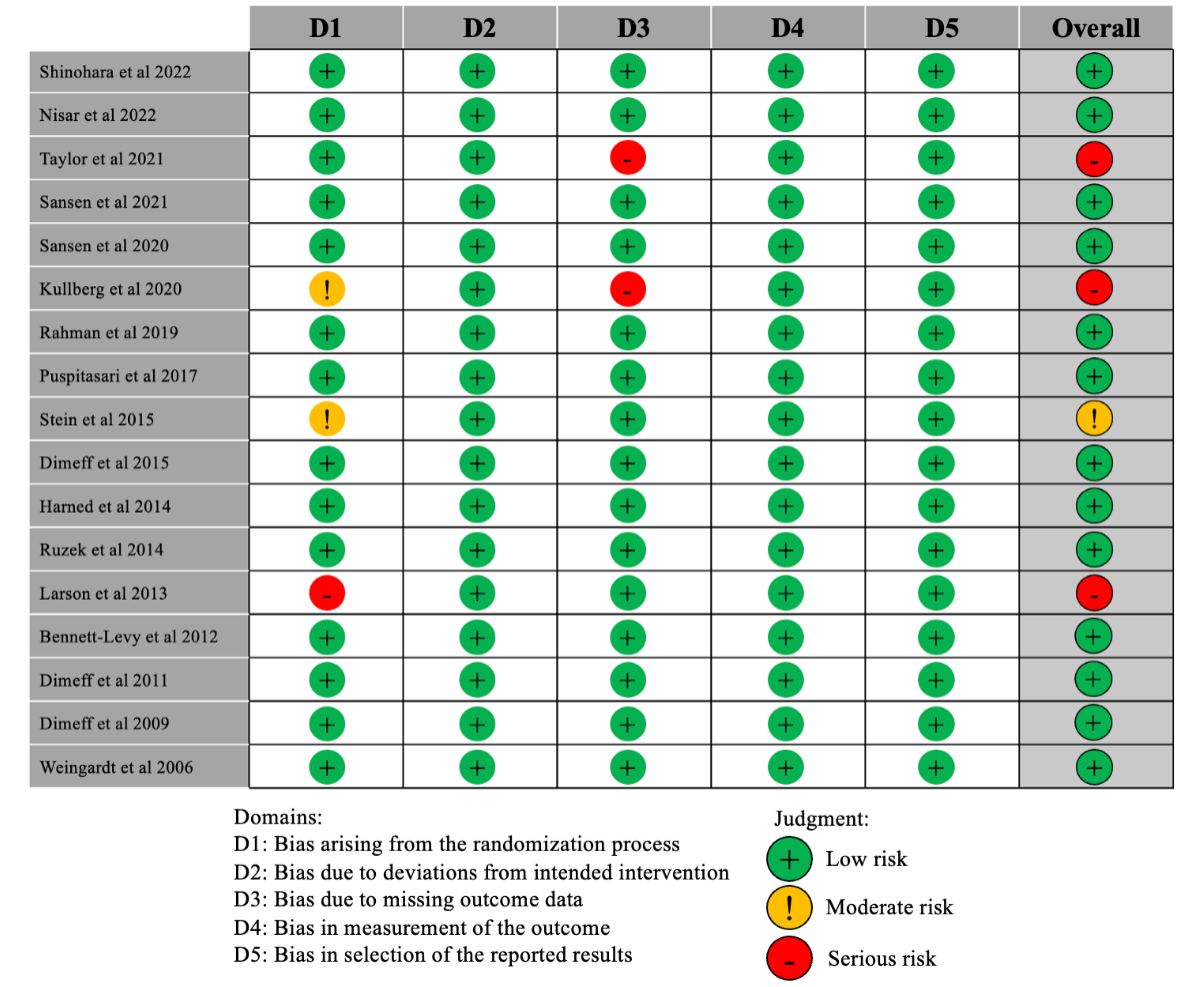
Risk of bias assessment for the randomized trials included in the review using the Cochrane risk of bias tool (RoB 2) [33-49].

Risk of bias was assessed both for the randomized and nonrandomized studies included in the review using the Cochrane risk of bias tools (RoB 2 and ROBINS-I). The risk of bias analysis was carried out by the first and last authors (KM, SES). The authors agreed upon the used tools and familiarized themselves with the logic behind them. The first author did the risk of bias analysis and classification. Then the last author examined the classification to identify potential discrepancies. No discrepancies occurred. In the review, 13 out of the 17 randomized studies, which corresponds to 76%, have been categorized as exhibiting a low risk of bias. One of the studies (6%) showed some concerns regarding the risk of bias. Three of the studies (17%) were flagged for having a high risk of bias. One of these studies had a source of bias from the randomization process and the 2 others have a potential source of bias from a large amount of missing outcome data. These results can be viewed in Figure 2. The ROBINS-I classification for risk of bias in nonrandomized studies included in the review can be viewed in Multimedia Appendix 1.

Among the nonrandomized studies included in the review, 16 out of 17 (94%) were flagged for having either a serious or critical risk of bias. The source of bias in all these studies was, at the very least, inadequate control of potential confounding variables. One study [58] was flagged at being only at moderate risk of bias.

### Learning Outcomes According to the Kirkpatrick Model Subcategories

#### Kirkpatrick: Reaction

Out of the 34 articles included in the review, 7 (21%) presented findings related to the “Reaction” level of the Kirkpatrick model. These findings included such concepts as satisfaction, acceptance, relevance, first impressions, viability, practicality, and feasibility conceptualized as observed barriers and facilitators regarding implementation of the trained content. E-Learning was associated with positive changes in these outcomes in training programs for dialectical behavior therapy (DBT) [48], CBT for psychosis [52], therapeutic crisis management [55], CBT for depression [62], prolonged exposure to posttraumatic stress disorder (PTSD) [59], and CBT for anxiety disorders [65].

#### Kirkpatrick: Learning

Out of the studies included in the review, 30 out of 34 (88%) presented findings related to the “Learning” level of the Kirkpatrick model. These included concepts related to acquisition of knowledge and skills, as well changes in attitudes and the level of self-efficacy. E-Learning was associated with positive changes in these outcomes in training programs for perinatal mental health assessment and empathic communication [33], evidence-based intervention in perinatal depression [34], CBT for insomnia [35], trauma-focused CBT [36], suicide prevention practices [38], behavioral activation [40], DBT [42,47,48], exposure therapy for anxiety disorders [43], CBT skills for traumatic stress [44], CBT [45,46,58], CBT for addictions [49], skills in engaging men in therapy [51], CBT addressed for patient with acute cancer [53], implementation intentions [54], therapeutic crisis management techniques [55], suicide prevention [67], sensory modulation approaches [57], interpersonal therapy [60], CBT for eating disorders [61], short-term psychodynamic therapy [63], management of PTSD [55], and motivational interviewing [66].

#### Kirkpatrick: Behavior

Out of the studies included in the review, 7 out of 34 (21%) reported results that can be classified to the “Behavior” level of the Kirkpatrick model which in all cases was reported as application of the trained content in clinical practice. E-Learning was generally associated with positive changes in these outcomes in training programs for interpersonal and social rhythm therapy for bipolar disorder [41], DBT [42], exposure therapy for anxiety disorders [43], CBT [46], and implementation intentions [54].

#### Kirkpatrick: Results

No studies included in this review display results that could be classified in the “Results” level of the Kirkpatrick model.

### Comparison Between e-Learning and Conventional Training Methods

Thirteen out of 34 (38%) studies, in which 9 were randomized trials [34,35,39-42,46,48,49] and 4 were nonrandomized [52,55,58,63], reported a comparison between a web-based training and training carried out by conventional methods. Of the 13 studies examined, 2 (15%) [42,48] reported results suggesting superiority (related to knowledge acquisition outcomes) for e-learning compared with conventional training. Meanwhile, 3 (23%) [40,42,52] indicated inferiority (concerning outcomes related to skills acquisition, satisfaction, self-efficacy, motivation, opportunity for active participation, and trainee-assessed professional benefit). In addition, 12 (92%) [34,35,39,41,42,46,48,49,52,55,58,63] found no significant difference between e-learning and conventional training methods, reporting equivalence (in terms of outcomes related to knowledge acquisition, skills acquisition, clinical application of the methods trained, self-efficacy, satisfaction, and acceptance rates) at posttraining evaluations. It is worth noting that studies reporting multiple outcomes could be included in more than 1 category.

**Figure 3:**
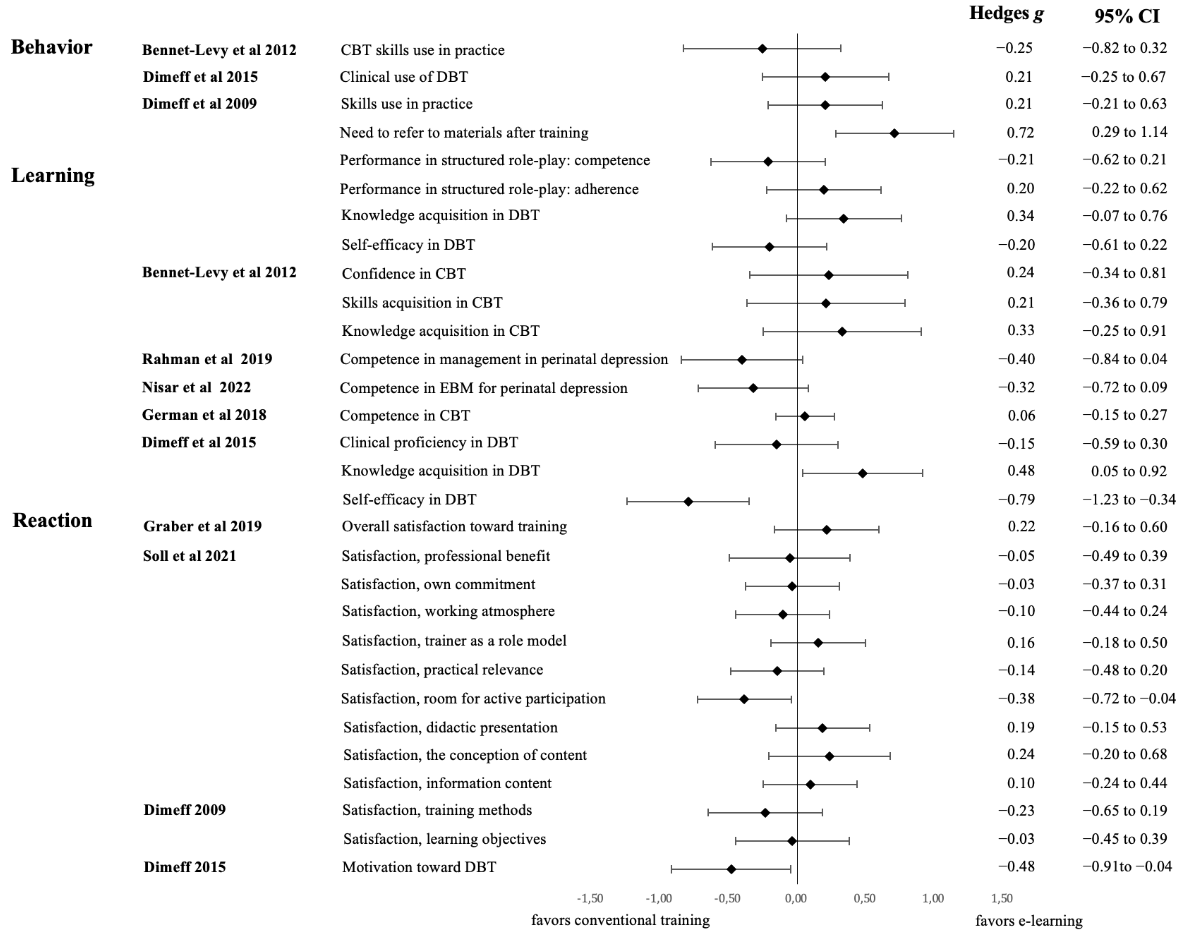
Forest plot of Hedges g effects sizes depicting the difference between e-learning and conventional training methods in Kirkpatrick levels 1-3 [34,39,42,46,48,52,55,58]. CBT: cognitive behavioral therapy; DBT: dialectical behavior therapy; EBM: evidence-based method.

Eight out of these 13 studies included the necessary figures to calculate Hedges *g*. The first authors of the remaining studies were contacted, but none provided the figures or raw data within an acceptable time window of 1 month. The overall mean effect size from all comparisons within the 8 studies (n=30) was 0.01. A forest plot displaying effect sizes and their corresponding 95% CIs is shown in Figure 3. The results are organized and presented in accordance with the Kirkpatrick model categorization.

## Discussion

### Principal Results

A systematic literature review of e-learning in the context of psychotherapy training was conducted following the PRISMA guidelines. E-Learning was positively associated with learning outcomes in terms of knowledge and skills acquisition as well as implementing learned skills to practice according to the Kirkpatrick levels 1-3. There were no apparent differences between e-learning and conventional training methods. None of the studies examined the impact of training on patient outcomes, and the research demonstrated significant heterogeneity.

This is the first study to exclusively assess the level of research regarding e-learning within this domain. The review included 34 studies conducted between 2008 and 2022, encompassing a mix of both randomized and nonrandomized research. Up to this point, findings on training outcomes have been reported across various outcomes and the level of evidence is still emerging. The target group, training content, a description of e-learning, sample sizes, primary outcomes, and a summary of results were provided from each article included in the review. Most of the randomized studies were linked with a low risk of bias, whereas nonrandomized studies exhibited a severe risk of bias. The risk of bias in the majority of the nonrandomized studies stemmed from limited control of confounding variables. The execution method of e-learning, the measurement methods used in training outcome assessments, and the trainees were highly heterogenous across the included studies. The high amount of heterogeneity in various aspects of the included literature and small sample sizes in many of the included studies create challenges in synthesizing the findings and drawing clear conclusions. Therefore, it is necessary to process the literature using a higher-lever categorization in learning outcomes (such as the Kirkpatrick model).

e-Learning within psychotherapy training showed positive associations with several aspects: trainee satisfaction, enhanced learning outcomes—including knowledge and skills acquisition, and the application of trained methods into practice, as observed in within-trainee designs. These findings align with levels 1-3 of the Kirkpatrick model. No outcomes related to patient outcomes were reported (Kirkpatrick level 4). The results of this review point in the direction of equivalence between e-learning and conventional training methods such as lectures and workshops. Training outcomes including trainee satisfaction, knowledge, and skill acquisition, and application of trained content in clinical work were comparable between e-learning and conventional training. The overall effect size signifying this difference was 0.01, indicating no difference.

In the only study that reported superiority of conventional training methods in skills acquisition e-learning involved solely watching previously recorded lectures rather than actively interacting with content specifically designed as an e-learning program [40]. In the other studies, where the inferiority of e-learning was presented as a result with a more modern approach on e-learning, the inferiority was in the context of trainee satisfaction, self-efficacy, and motivation [42,52]. E-Learning was reported to be superior compared with conventional training only in the context of knowledge acquisition when training DBT [42,48].

The trainees and the clinical contexts of the trainees varied heavily between included studies. Furthermore, the operationalization of the learning outcomes and the associated measurement methods were not consistent and included both subjective self-ratings of knowledge and competence and more objective measures (eg, quizzes and expert-rated competence scores). In most of the studies, the training content included CBT-based methods, and in some studies interpersonal therapy and short-term psychodynamic therapy. Most of the e-learning solutions described were similar and included case vignettes, demonstrations, multimedia didactic content, and various assignments.

### Strengths and Limitations

This review has many strengths. These include following the PRISMA guidelines, giving an exhaustive look in the current literature, and that the results of research comparing e-learning and conventional training methods were uniformly aggregated behind a single statistic. As anticipated, studies identified in this review had high heterogeneity of learning methods, therapy content, and outcomes, making systematic comparison difficult. We tried to control this heterogeneity by using the Kirkpatrick model of training evaluation, summarizing the results by calculating effect sizes, and requesting original data from authors to do that. However, it seems that heterogeneity is a feature of this field of study and cannot be easily overcome with post hoc methods; a more uniform research methodology would be needed in future studies.

This systematic review also has limitations. First, the review did not include gray literature (materials and research produced outside of academic publishing) or literature beyond the scope of the conducted search, which may include information relevant to this study. Second, in addition to the risk of bias assessment, the quality of the research included was not systematically assessed (eg, publication bias). Third, the search method yielded a substantial amount of literature not relevant to the study, which makes the replication of this review challenging. This is mainly attributed to the overlap in vocabulary between literature related to training and psychosocial interventions themselves. This issue also touches upon a possible methodological limitation in our screening process, namely, that the initial screening phases were conducted by a sole reviewer. The purpose of this preliminary screening was to promptly identify and eliminate studies that were unmistakably irrelevant to our review, with a large proportion of such studies investigating digital psychosocial interventions (eg, internet-based cognitive behavioral therapy), a testament to the prevalent overlap in terminologies used. To ensure a balanced review, the authors applied a statistical approach to select a representative sample of studies for evaluation by the second author. We posit that the inclusion of 2 independent raters from the outset, as opposed to 1, during the initial screening phases might have had a negligible effect on the outcomes, considering that all studies with any element of ambiguity were carried forward for full review. Nonetheless, we acknowledge that our approach may be more susceptible to bias compared with methodologies that use multiple independent reviewers for the assessment of all studies. We consider this as a potential limitation and agree that the use of dual raters throughout the process might augment the robustness against bias.

### Conclusions

Based on this literature review, we conclude that positive learning outcomes are generally associated with various e-learning programs in psychotherapy training including trainee satisfaction, knowledge and skill acquisition, and in application of trained content in clinical practice. Learning outcomes are generally equivalent between e-learning and conventional training methods. This equivalence is interpreted as a supportive finding for e-learning, considering the other benefits of e-learning, such as scalability and consistency of quality. While more research is warranted, and research methodology needs to be more consistent in the description of e-learning solutions and in learning outcome assessment, it seems advisable to use e-learning as a part of psychotherapy training curriculums.

e-Learning creates an opportunity to scale up training in large and even in nationwide settings. This, in turn, can significantly improve equal and sufficient access to psychotherapy in public health care settings, which are impossible with conventional and unscalable training methods [61]. This can be seen, for example, within the study by Wilkerson et al [50], where it was estimated that 2500 individuals will annually complete an e-learning program in Cognitive Behavioral Therapy for Insomnia, a scale the authors declared as “massive dissemination.” With centralized digital solutions, e-learning might prove itself as the more cost-effective solution than conventional training methods while ensuring quality in training as many learning outcomes are comparable between these methods.

As the literature is still emerging and current corpus is displaying a high amount of heterogeneity, small sample sizes, and inconsistent description of e-learning solutions and measurement methods in learning outcomes, it would be advisable to use e-learning as a part of blended training programs. By comparison with e-learning results in health education in general, a blended training model might even be the most optimal way to implement e-learning. E-Learning is generally recognized as an opportunity to improve the availability of training programs by eliminating constraints set by time and geographical location and also a way to increase the quality of training programs by shifting the focus of the surrounding pedagogics toward more learning-centric direction. Knowledge acquisition can be supported through e-learning, but refining skills and ensuring competencies may require synchronous interaction with a trainer to reach optimal learning outcomes [67].

In psychotherapy training, a shift toward minimizing the didactic approach of trainers by facilitating the active role of the trainee in knowledge acquisition is warranted. E-Learning viewed as a pedagogy rather than technology may be an optimal solution in implementing flipped classroom and other activating learning methods. Within these methods, synchronous learning events are held with trainees who have already familiarized themselves with the necessary materials and focus on honing therapeutic and reflective skills and addressing student motivation. Future research needs to focus on replicating the effects of e-learning in trainee satisfaction, learning and behavior, and on studying the effectiveness and cost-effectiveness of training (e-learning or conventional methods) on patient outcomes. Research is especially needed to further determine the optimal way to combine e-learning and conventional training methods. Furthermore, to reach the full potential of e-learning in the context of psychotherapy training, international collaboration is warranted to bring consistency on how e-learning systems are developed, described, and assessed.

## Appendix

Multimedia Appendix 1 ROBINS-I (Risk of Bias in Non-randomized Studies of Interventions) classification for risk of bias in nonrandomized studies included in the review.

Multimedia Appendix 2 PRSIMA 2020 checklist.

## Abbreviations

CBT: cognitive behavioral therapy
DBT: dialectical behavior therapy
PRISMA: Preferred Reporting Items for Systematic Reviews and Meta-Analyses
PTSD: posttraumatic stress disorder
ROBINS-I: Risk of Bias in Non-randomized Studies of Interventions

## Data Availability

The data extracted from the included studies are available upon request from the first author.
