# Supplementary figures and images for: Learning Outcomes of e-Learning in Psychotherapy Training and Comparison With Conventional Training Methods: Systematic Review
Source: J Med Internet Res. 2024 Jul 29;26:e54473. doi: 10.2196/54473 (PMC11319893; doi:10.2196/54473)

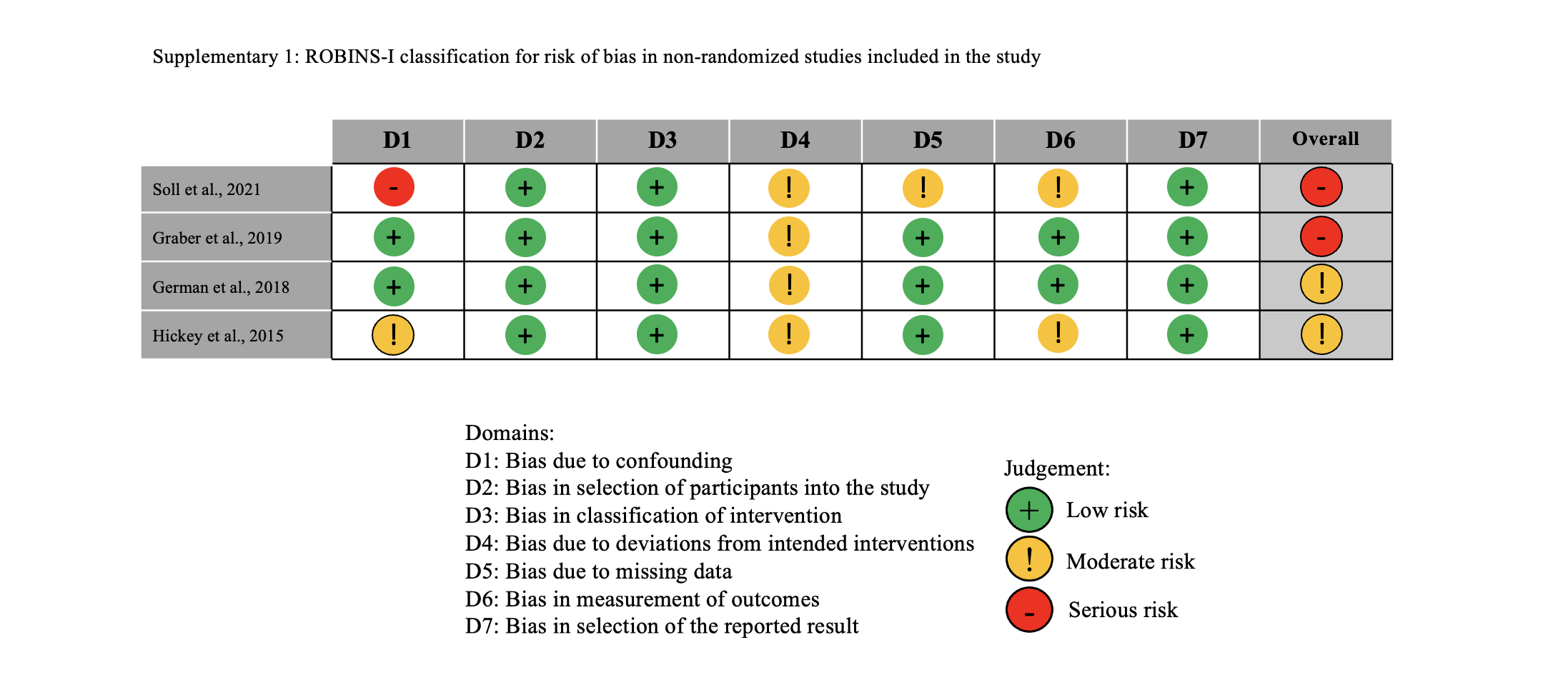

Supplement: Multimedia Appendix 1 [file jmir_v26i1e54473_app1.docx]
